# Material-dependent chromatic recovery of coffee-stained resin composites after in-office hydrogen peroxide bleaching

**DOI:** 10.4317/jced.64520

**Published:** 2026-09-15

**Authors:** Andrea Lucia Méndez-Gil, José Giancarlo Tozo-Burgo, Roger Calla-Poma, Marco Sánchez-Tito

**Affiliations:** 1 Private Practice, Tacna, Perú; 2 Research Group on Dental Biomaterials and Natural Products, Faculty of Health Sciences, Universidad Privada de Tacna, 23000, Tacna, Perú; 3 Faculty of Dentistry, Universidad Nacional Mayor de San Marcos, 15001 Lima, Perú

**Keywords:** Color stability, resin composites, nanohybrid composites, CIEDE2000, coffee staining, hydrogen peroxide, dental biomaterials.

## Abstract

**Background:**

This study evaluated the chromatic recovery of two resin composites after staining with different coffee preparations and subsequent treatment with a 35% hydrogen peroxide bleaching agent.

**Materials and Methods:**

Ninety disc-shaped specimens of Filtek™ Z350 XT and IPS Empress® Direct were distributed into six groups (n=15) according to staining medium: Instant coffee, brewed coffee, or distilled water. After 8 days of immersion, specimens were treated with 35% hydrogen peroxide. Color was measured spectrophotometrically, and CIEDE2000 (ΔE00) differences were calculated between baseline and post-staining (T1), post-staining and post-bleaching (T2), and baseline and post-bleaching (T3). Data were analyzed using non-parametric tests and a generalized linear model (α=0.05).

**Results:**

Coffee produced greater color variation than distilled water in both composites. Bleaching promoted partial chromatic recovery, although residual differences from baseline remained. Post-bleaching ΔE00 differed significantly among staining media (p<0.05), with instant coffee producing the greatest variation. IPS Empress® Direct showed higher post-bleaching ΔE00 values than Filtek™ Z350 XT (p<0.001), with no significant resin-staining medium interaction (p=0.267). All residual color differences were below the acceptability threshold (ΔE00=1.8).

**Conclusions:**

Coffee caused perceptible discoloration in both composites, while 35% hydrogen peroxide bleaching reduced but did not completely reverse these changes. Chromatic recovery depended on the restorative material and coffee preparation under the evaluated experimental conditions.

## Introduction

The increasing demand for aesthetic dental treatments has intensified the clinical use of resin-based restorative materials and tooth-whitening procedures [1,2]. However, despite the favorable optical properties of contemporary resin composites, discoloration remains one of the main causes of aesthetic deterioration and restoration replacement over time [3]. The color stability of resin composites is influenced not only by extrinsic factors, such as exposure to pigmented beverages, but also by intrinsic material-related characteristics including resin matrix composition, filler content, degree of conversion, water sorption, and surface degradation [4].

Among dietary chromogenic agents, coffee is considered one of the beverages with the greatest staining potential for resin-based materials [5,6]. Coffee contains a variety of chromogenic compounds that can interact with the resin matrix through adsorption and absorption, contributing to progressive discoloration of resin composites [3,7]. Previous studies have shown that the extent of discoloration depends on the physicochemical characteristics of the restorative material, particularly the hydrophilicity of the resin matrix, the type of monomers, filler particle distribution, and the integrity of the filler-matrix interface [7-9]. Resin matrices containing hydrophilic monomers, such as Bis-GMA and TEGDMA, tend to exhibit greater water sorption and increased susceptibility to pigment diffusion than matrices enriched with more hydrophobic monomers such as UDMA [10].

The optical behavior of resin composites after staining may also vary according to the composition and preparation method of the staining medium. Variations in roasting process, extraction method, concentration of soluble compounds, and pigment composition can modify the staining potential of coffee beverages [11]. Although several studies have evaluated discoloration induced by common beverages, the influence of different coffee preparations on subsequent chromatic recovery after bleaching remains less explored.

Hydrogen peroxide-based bleaching agents have traditionally been used to oxidize chromogenic molecules and reduce discoloration in dental tissues [12]. The oxidative degradation of complex chromophores into smaller and less light-absorbing molecules may also influence the optical properties of stained resin composites [13]. Nevertheless, the bleaching response of restorative materials remains inconsistent and appears to be material-dependent. Previous studies have demonstrated that bleaching procedures may partially reduce discoloration in composite resins without completely restoring baseline color values [14-16]. Moreover, differences in photoinitiator systems, resin matrices, filler technology, and water sorption behavior may influence the susceptibility of composite resins to both staining and post-bleaching chromatic recovery [17,18].

Filtek™ Z350 XT and IPS Empress® Direct are resin composites with distinct organic matrices and photoinitiator systems that may exhibit different optical responses after exposure to staining and oxidative agents. Although staining and subsequent bleaching of resin composites have been previously investigated, the combined influence of restorative material and coffee preparation on post-bleaching chromatic recovery remains less explored. Therefore, the aim of this study was to evaluate the effect of a 35% hydrogen peroxide in-office bleaching agent on the color stability and chromatic recovery of two nanohybrid resin composites previously stained with two different coffee preparations.

## Materials and Methods

*Study design and sample size calculation*

This *in vitro* experimental study was conducted following approval from an institutional research ethics committee. The sample size was calculated using G*Power software version 3.1.9.4 (Heinrich Heine University, Düsseldorf, Germany). The calculation considered an effect size of 0.45, a statistical power of 80%, and a significance level of 5%, resulting in a minimum total sample size of 90 specimens distributed across six experimental groups (n=15 per group). Two resin composites were evaluated: Filtek™ Z350 XT (3M ESPE, St. Paul, MN, USA) and IPS Empress® Direct (Ivoclar Vivadent, Schaan, Liechtenstein), both in shade A2 enamel. The composition and characteristics of the evaluated materials are presented in Table 1. Specimens were randomly allocated according to resin type and staining medium. Specimens presenting surface defects, voids, marginal irregularities, incomplete polymerization, or visible polishing damage were excluded prior to color analysis and replaced to maintain the predetermined sample size of 15 specimens per group. Outcome measurements were performed by a single calibrated operator; blinding to the experimental groups was not implemented.

*Sample preparation*

Disc-shaped specimens (8 mm diameter x 2 mm thickness) were fabricated using a standardized stainless-steel mold. The resin composite was inserted in a single increment and covered with a polyester strip and glass slide to obtain a flat and standardized surface [19]. Specimens were light-cured using a VALO™ LED curing unit (Ultradent Products Inc., South Jordan, UT, USA) for 20 s at a nominal irradiance of 1200 mW/cm², according to the manufacturer’s recommendations [20]. After polymerization, all specimens were stored in distilled water at 37°C for 24 h to allow post-cure stabilization before finishing and polishing procedures. Specimen surfaces were subsequently polished using Sof-Lex™ aluminum oxide discs (3M ESPE, St. Paul, MN, USA) in a sequential order from coarse to superfine grit under standardized dry conditions for 30 s using a low-speed handpiece (FX205-B2, NSK-Nakanishi Inc., Kanuma, Tochigi, Japan) [21]. After polishing, specimens were rinsed with distilled water and air-dried. Surface quality was visually inspected under standardized lighting conditions to confirm the absence of defects, marginal irregularities, or polishing damage before color assessment.

*Baseline color measurement*

Baseline color measurements were obtained using an Easyshade V spectrophotometer (VITA Zahnfabrik, Bad Säckingen, Germany). Before each measurement session, the device was calibrated according to the manufacturer’s instructions. Each specimen was positioned on a standardized white non-reflective background to minimize optical interference during color assessment. Three consecutive measurements were obtained from the central region of each specimen, and the mean CIE L*, a*, and b* coordinate values were recorded for analysis. To reduce measurement variability, all color assessments were performed by a single calibrated operator under standardized ambient lighting conditions. Specimens were cleaned and gently air-dried before each measurement procedure.

*Staining process*

Two coffee preparations were used as staining media: Instant coffee (Altomayo Clásico, Cajamarca, Peru) and brewed coffee (Occobambino®, Quillabamba, Cusco, Peru). The instant coffee solution was prepared according to previously described staining protocols by dissolving coffee powder in boiling distilled water immediately before use [9]. Brewed coffee was prepared using an automatic coffee machine (De'Longhi Magnifica Evo, De'Longhi, Treviso, Italy) according to a previously described protocol [22]. Specimens were individually immersed in 5 mL of the corresponding medium using 24-well culture plates. The staining solutions were maintained at room temperature and renewed daily throughout the experimental period to minimize chemical degradation and maintain staining consistency. The immersion procedure lasted 8 days, representing a theoretical cumulative exposure equivalent to approximately 1.5 years of coffee consumption, based on an estimated exposure time of 20 min per cup [23]. Control specimens were stored in distilled water under identical conditions throughout the experimental period.

*Post-exposure color measurement*

After completion of the staining protocol, specimens were rinsed under running distilled water for 1 min to remove superficial residues and gently dried with absorbent paper. Color measurements were subsequently performed using the same spectrophotometric protocol described for baseline assessment. Three consecutive measurements were obtained from the central region of each specimen under standardized lighting conditions, and the mean CIE L*, a*, and b* values were recorded for analysis.

*Bleaching procedure*

Following the 8-day exposure period, all specimens, including those exposed to both coffee preparations and distilled water, were treated with a 35% hydrogen peroxide in-office bleaching agent (Whiteness HP Maxx, FGM Dental Products, Joinville, Brazil). The bleaching gel was applied uniformly over the specimen surface for 15 min according to the manufacturer’s instructions, followed by rinsing with distilled water for 1 min. This procedure was repeated three consecutive times for each specimen. After completion of the bleaching protocol, specimens were stored in distilled water at 37°C until the final color assessment. To ensure procedural standardization, the same operator performed all bleaching applications under controlled laboratory conditions.

*Color change evaluation*

Color measurements were performed after completion of the bleaching procedure using the same spectrophotometric protocol previously described. Color differences were calculated for three measurement intervals:

T1: Color difference between baseline and post-staining measurements.

T2: Color difference between post-staining and post-bleaching measurements.

T3: Color difference between baseline and post-bleaching measurements.

The color difference between the measurements was assessed using the CIEDE2000 color difference formula [24], where ΔL′, ΔC′, and ΔH′ represent differences in lightness, chroma, and hue, respectively:


$${\mathrm{\Delta E}}_{00}={\left[{\left(\frac{\Delta L}{K_{L}S_{L}}\right)}^{2}+{\left(\frac{\Delta C}{K_{C}S_{L}}\right)}^{2}+{\left(\frac{\Delta H}{K_{H}S_{H}}\right)}^{2}+R_{T}\,(\frac{\Delta C}{K_{C}S_{C}})\,(\frac{\Delta H}{K_{H}S_{H}})\right]}^{\frac{1}{2}}$$

The term RT corresponds to the rotation function that improves chromatic difference prediction in the blue region. The weighting functions S_L_, S_C_, and S_H_ adjust the contributions of lightness, chroma, and hue according to visual perception parameters. In the present study, parametric factors were set as k_L_=k_C_=k_H_=1 [24]. The obtained ΔE_00_ values were interpreted according to the 50:50% perceptibility threshold (PT=0.8) and acceptability threshold (AT=1.8) recommended for resin-based restorative materials [25].

*Statistical analysis*

Statistical analysis was performed using Stata® version 18.0 (StataCorp LP, College Station, TX, USA). Descriptive statistics included means, standard deviations, medians, interquartile ranges, and minimum and maximum values according to data distribution characteristics. For comparisons of post-bleaching ΔE_00_ values among staining media within each resin composite, normality and homoscedasticity were assessed from the model residuals using the Shapiro-Wilk and Breusch-Pagan/Cook-Weisberg tests, respectively. As these assumptions were not satisfied, differences in post-bleaching ΔE_00_ values among staining media within each resin composite were evaluated using the Kruskal-Wallis test, followed by pairwise comparisons with Bonferroni adjustment. To evaluate the effects of resin composite and staining medium on post-bleaching color variation, a generalized linear model with Gaussian family, identity link function, and robust variance estimator was constructed. The model included resin composite, staining medium, and their interaction term. This approach allowed simultaneous estimation of the effects of both experimental factors and their interaction, with adjusted effect estimates and 95% confidence intervals. Robust variance estimation was used to reduce the influence of heteroscedasticity on statistical inference. Graphical representations were generated using GraphPad Prism® version 10.0 (GraphPad Software Inc., San Diego, CA, USA). A significance level of 5% was adopted for all statistical analyses.

## Results

Table 2 summarizes the ΔE_00_ values according to resin composite, staining medium, and measurement interval. T1 represents the color difference induced by exposure, T2 the color difference associated with bleaching, and T3 the residual color difference between baseline and post-bleaching measurements. Coffee exposure produced greater color variation in both resin composites than distilled water at T1. Following bleaching, substantial color changes were observed between post-staining and post-bleaching measurements (T2), indicating a bleaching-related chromatic response. At T3, residual color differences were considerably lower than the initial coffee-induced differences observed at T1, indicating partial chromatic recovery toward baseline. Residual T3 values remained above the perceptibility threshold (PT=0.8) in both coffee groups for Filtek™ Z350 XT and in the Altomayo coffee group for IPS Empress® Direct, whereas all T3 values remained below the acceptability threshold (AT=1.8).

Post-bleaching color differences according to staining medium are presented in Table 3. Significant differences in ΔE_00_ values were observed among staining media within each resin composite (*p*<0.05). Altomayo coffee produced the highest post-bleaching color variation, whereas distilled water showed the lowest ΔE_00_ values in both materials.

The generalized linear model (Table 4) demonstrated that both resin composite and staining medium significantly influenced post-bleaching ΔE_00_ values. IPS Empress® Direct exhibited significantly higher color variation than Filtek™ Z350 XT, while both coffee preparations produced significantly greater ΔE_00_ values than distilled water (*p*<0.001). No significant interaction was observed between resin composite and staining medium (*p*=0.267).

The distribution of ΔE_00_ values according to resin composite, staining medium, and evaluation stage is shown in Figure 1.

## Discussion

The present study evaluated the chromatic recovery behavior of two nanohybrid resin composites after exposure to different coffee preparations and subsequent treatment with a 35% hydrogen peroxide bleaching agent. Both resin composites exhibited perceptible color changes after coffee immersion, followed by a reduction in ΔE_00_ values after bleaching. However, post-bleaching color differences remained detectable, indicating that oxidative treatment promoted only partial chromatic recovery rather than complete restoration to baseline color. Although staining and bleaching of resin composites have been previously investigated, the present study extends this evidence by simultaneously comparing two coffee preparations and two resin composites under the same bleaching protocol, allowing the influence of both the restorative material and staining medium on chromatic recovery to be evaluated.

Discoloration of resin-based materials is a multifactorial phenomenon associated with the interaction between the organic resin matrix, filler characteristics, water sorption, and the chemical composition of staining agents [3,7-10]. Coffee has been consistently identified as one of the beverages with the greatest staining potential for resin-based materials [5,6]. In the present study, both coffee preparations produced substantially greater ΔE_00_ values than distilled water, confirming the strong staining susceptibility of nanohybrid resin composites exposed to coffee-based beverages.

The staining behavior observed in the present study may also be associated with differences in the composition of the evaluated resin composites. Filtek™ Z350 XT demonstrated lower post-bleaching ΔE_00_ values than IPS Empress® Direct, suggesting greater chromatic recovery after oxidative treatment. The optical stability of resin composites is strongly influenced by the hydrophilicity of the organic matrix and the physicochemical characteristics of the polymer network. Previous studies have shown that increased water sorption may facilitate pigment diffusion and promote discoloration of resin-based materials [3,10]. Hydrophilic monomers such as Bis-GMA and TEGDMA exhibit greater affinity for water uptake, which may plasticize the polymer matrix and favor penetration of chromogenic compounds into the resin structure [10]. However, because water sorption and monomer-specific behavior were not directly evaluated in the present study, these mechanisms should be regarded as possible explanations rather than direct evidence for the differences observed between the materials.

In addition to monomer composition, filler characteristics and the integrity of the filler-matrix interface may influence optical degradation. Water sorption can contribute to degradation at the filler-matrix interface and potentially facilitate optical alterations [3]. Hydrolytic degradation may also contribute to long-term material degradation through deterioration of the silane coupling layer [26]. Nevertheless, no microstructural or physicochemical analyses were performed in the present study; therefore, the contribution of these mechanisms to the differences between Filtek™ Z350 XT and IPS Empress® Direct cannot be established from the present findings.

Differences in photoinitiator systems may also have contributed to the distinct chromatic responses observed between the evaluated materials. IPS Empress® Direct incorporates Lucirin-based photoinitiators, whereas Filtek™ Z350 XT primarily relies on camphorquinone-based systems. Lucirin-containing composites have been associated with differences in color stability compared with conventional photoinitiator systems [27,28]. However, photoinitiator-related effects were not independently assessed in this study, and their contribution to the observed material-dependent response remains hypothetical.

The type of coffee preparation also significantly influenced post-bleaching color variation. Altomayo coffee produced higher ΔE_00_ values than Occobambino coffee in both resin composites. Coffee composition may vary according to bean origin, roasting process, extraction method, concentration of soluble compounds, and chemical composition [11]. These differences could potentially influence staining behavior; however, the chemical composition and chromogenic content of the two coffee preparations were not analyzed. Therefore, the greater color variation associated with Altomayo coffee cannot be attributed to a specific chemical component or mechanism.

Hydrogen peroxide-based bleaching agents act through oxidative degradation of chromogenic molecules into smaller compounds with lower light absorption capacity [12,13]. Although bleaching produced substantial color changes relative to the post-staining condition, complete recovery to baseline color was not achieved. These findings are consistent with previous studies demonstrating that bleaching procedures may reduce discoloration without fully restoring the original color of resin-based materials [14-16]. Similarly, Hussain *et al*. reported that bleaching partially improved the color of coffee-stained resin composites without restoring their original optical appearance [29]. When the residual color difference relative to baseline (T3) was considered, values remained above the perceptibility threshold (PT=0.8) for both coffee groups in Filtek™ Z350 XT and for the Altomayo group in IPS Empress® Direct. Nevertheless, all T3 values remained below the acceptability threshold (AT=1.8). Thus, statistically significant differences among groups should not be interpreted as necessarily representing clinically unacceptable color differences.

The generalized linear model demonstrated that both resin composite and staining medium independently influenced post-bleaching ΔE_00_ values, whereas the interaction between these variables was not statistically significant. This finding suggests that the effect of the staining medium on chromatic recovery followed a similar pattern in both resin composites, despite differences in the magnitude of color variation. The results indicate a material-dependent bleaching response and an independent contribution of coffee preparation to post-bleaching color change.

The present study has limitations inherent to its *in vitro* design. The experimental model did not reproduce important clinical variables such as salivary pellicle formation, toothbrushing abrasion, thermal cycling, pH fluctuations, dietary variability, and long-term aging conditions, all of which may influence the optical behavior of resin composites. Moreover, continuous immersion for 8 days represents a cumulative laboratory exposure and does not reproduce the intermittent pattern of coffee consumption under oral conditions. The study was also limited to two resin composites, two coffee preparations, and a single 35% hydrogen peroxide bleaching protocol, which restricts the generalizability of the findings.

Additionally, no surface characterization analyses, such as scanning electron microscopy or surface roughness evaluation, were performed to investigate possible microstructural alterations associated with staining and bleaching procedures. Neither water sorption nor the chemical composition of the coffee preparations was directly assessed, limiting mechanistic interpretation of the observed differences. Future studies should incorporate clinically realistic exposure cycles, additional restorative materials and bleaching protocols, repeated bleaching procedures, repolishing strategies, artificial aging protocols, and complementary physicochemical analyses as well as *in vivo* investigations to better understand the mechanisms underlying discoloration and chromatic recovery in resin-based restorative materials.

## Conclusions

Coffee exposure promoted clinically perceptible discoloration in both nanohybrid resin composites, while bleaching with 35% hydrogen peroxide reduced, but did not completely reverse, the observed color changes. Residual color differences after bleaching were below the clinical acceptability threshold for all groups. Filtek™ Z350 XT exhibited greater chromatic recovery and lower post-bleaching ΔE_00_ values than IPS Empress® Direct. In addition, the staining potential and post-bleaching optical response varied according to the coffee preparation, suggesting that discoloration and chromatic recovery are influenced by both the restorative material and the staining medium. These conclusions are limited to the two resin composites, coffee preparations, and 35% hydrogen peroxide bleaching protocol evaluated in this study. 

## Figures and Tables

**Table 1:** Table 1: Composition and characteristics of the evaluated resin composites.

**Composite**	**Shade**	**Manufacturer**	**Type**	**Organic matrix**	**Inorganic filler (% wt / % vol)**	**Particle size**
Filtek™ Z350 XT	A2	3M ESPE, St. Paul, MN, USA	Nanofilled resin composite	Bis-GMA, UDMA, TEGDMA, PEGDMA, Bis-EMA	Non-agglomerated silica (20 nm), non-agglomerated zirconia (4-11 nm), and aggregated zirconia/silica nanoclusters; total inorganic filler 78.5 wt% (63.3 vol%)	4-20 nm; nanoclusters up to 0.6-1.4 μm
IPS Empress® Direct	A2	Ivoclar Vivadent AG, Schaan, Liechtenstein	Nanohybrid resin composite	Bis-GMA, UDMA, DCP, Bis-PMA	Barium glass, mixed Si-Zr oxide, and barium-aluminum fluorosilicate glass; total inorganic filler 52-59 vol%	0.03-16.3 μm

**Table 2:** Table 2: Descriptive statistics of ΔE_00_ values according to resin composite, staining medium, and evaluation stage.

**ΔE00**	**Resin composite**	**Staining medium**	**Mean ± SD**	**Median [IQR]**	**Min**	**Max**
T1	Filtek™ Z350 XT	Altomayo coffee	4.32 ± 0.68	4.47 [1.20]	3.33	5.25
		Occobambino coffee	3.08 ± 1.07	2.85 [1.44]	1.45	5.11
		Distilled water	0.18 ± 0.13	0.13 [0.14]	0.04	0.52
	IPS Empress® Direct	Altomayo coffee	4.91 ± 0.52	4.99 [0.72]	3.88	5.64
		Occobambino coffee	3.53 ± 0.90	3.55 [1.19]	2.03	5.50
		Distilled water	0.09 ± 0.03	0.10 [0.04]	0.05	0.15
T2	Filtek™ Z350 XT	Altomayo coffee	3.76 ± 0.61	3.82 [1.23]	2.97	4.68
		Occobambino coffee	2.53 ± 0.93	2.39 [1.06]	1.42	4.27
		Distilled water	0.79 ± 0.30	0.75 [0.27]	0.24	1.47
	IPS Empress® Direct	Altomayo coffee	4.37 ± 0.41	4.42 [0.71]	3.74	5.09
		Occobambino coffee	3.61 ± 0.78	3.43 [0.97]	2.44	5.70
		Distilled water	0.51 ± 0.27	0.52 [0.41]	0.12	1.13
T3	Filtek™ Z350 XT	Altomayo coffee	0.85 ± 0.36	0.79 [0.35]	0.44	1.79
		Occobambino coffee	0.94 ± 0.40	0.84 [0.54]	0.45	1.86
		Distilled water	0.75 ± 0.26	0.78 [0.32]	0.19	1.31
	IPS Empress® Direct	Altomayo coffee	0.93 ± 0.29	0.90 [0.33]	0.59	1.63
		Occobambino coffee	0.66 ± 0.39	0.63 [0.44]	0.08	1.73
		Distilled water	0.50 ± 0.28	0.41 [0.31]	0.10	1.12

T1: Color difference between baseline and post-staining measurements. T2: Color difference between post-staining and post-bleaching measurements. T3: Color difference between baseline and post-bleaching measurements. SD: Standard deviation; IQR: Interquartile range.

**Table 3:** Table 3: Post-bleaching color coordinates and ΔE_00_ values according to resin composite and staining medium.

**Resin composite**	**Staining medium**	**Δ** *L* ** Median [IQR]**	**Δ** *a* ** Median [IQR]**	**Δ** *b* ** Median [IQR]**	**ΔE00** **Median [IQR]**
Filtek™ Z350 XT	Altomayo coffee	5.00 [1.33]	-0.93 [0.53]	-2.66 [1.03]	3.82 [1.43]ᵃ
	Occobambino coffee	2.93 [1.36]	-0.53 [0.36]	-2.40 [0.96]	2.39 [1.15]ᵇ
	Distilled water	0.67 [0.28]	-0.10 [0.09]	-0.43 [0.21]	0.75 [0.27]ᶜ
IPS Empress® Direct	Altomayo coffee	5.30 [0.83]	-1.23 [0.40]	-3.86 [2.00]	4.42 [0.77]ᵃ
	Occobambino coffee	4.10 [1.03]	-1.10 [0.66]	-3.90 [2.00]	3.43 [1.00]ᵇ
	Distilled water	0.48 [0.31]	-0.05 [0.08]	-0.36 [0.24]	0.52 [0.41]ᶜ

*Values are expressed as median [interquartile range]. Different superscript lowercase letters indicate statistically significant differences between staining media within the same resin composite (Kruskal-Wallis test followed by pairwise comparison with Bonferroni adjustment, p<0.05).

**Table 4:** Table 4: Generalized linear model evaluating the effects of resin composite and staining medium on post-bleaching ΔE_00_ values.

**Variable**	**β coefficient**	**Robust SE**	*p * **value**	**95% CI**
Resin composite				
Filtek™ Z350 XT	Ref.	-	-	-
IPS Empress Direct	0.88	0.17	<0.001	0.54 to 1.22
Staining medium				
Distilled water	Ref.	-	-	-
Altomayo coffee	3.18	0.24	<0.001	2.71 to 3.66
Occobambino coffee	2.27	0.23	<0.001	1.81 to 2.73
Interaction term				
Resin composite × staining medium	0.31	0.28	0.267	-0.25 to 0.87

Ref.: Reference category; SE: Standard error; CI: Confidence interval.

**Figure 1:**
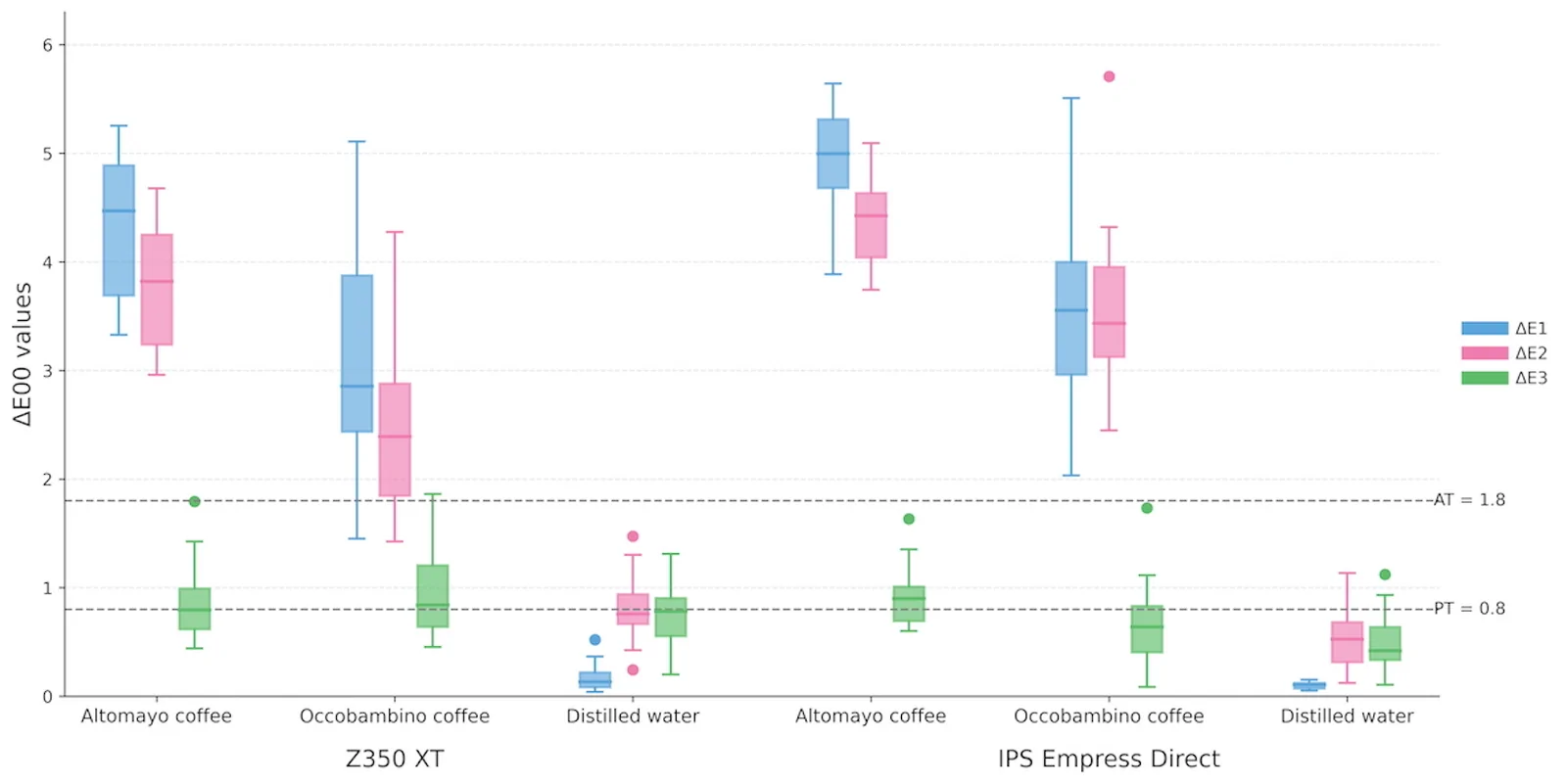
Fig. 1: Distribution of ΔE_00_ values according to resin composite, staining medium, and measurement interval. Dashed horizontal lines indicate the perceptibility threshold (PT=0.8) and acceptability threshold (AT=1.8).

## Data Availability

The datasets are available from the corresponding author upon reasonable request.
